# Implications of environmental and pathogen-specific determinants on clinical presentations and disease outcome in melioidosis patients

**DOI:** 10.1371/journal.pntd.0007312

**Published:** 2019-05-15

**Authors:** Tushar Shaw, Chaitanya Tellapragada, Asha Kamath, Vandana Kalwaje Eshwara, Chiranjay Mukhopadhyay

**Affiliations:** 1 Department of Microbiology, Kasturba Medical College, Manipal Academy of Higher Education, Manipal, Karnataka, India; 2 Department of Statistics, Prasanna School of Public Health, Manipal Academy of Higher Education, Manipal, Karnataka, India; Lowell General Hospital, UNITED STATES

## Abstract

**Background:**

Melioidosis is gaining recognition as an emerging infectious disease with diverse clinical manifestations and high-case fatality rates worldwide. However, the molecular epidemiology of the disease outside the endemic regions such as northeast part of Thailand and northern Australia remains unclear.

**Methodology/Principal findings:**

Clinical data and *B*. *pseudomallei* isolates obtained from 199 culture-confirmed cases of melioidosis diagnosed during 2006–2016 in South India were used to elucidate the host and pathogen specific variable virulence determinants associated with clinical presentations and disease outcome. Further, we determined the temporal variations and the influence of ecological factors on *B*.*pseudomallei* Lipopolysaccharide (LPS) genotypes causing infections. Severe forms of the disease were observed amongst 169 (85%) patients. Renal dysfunction and infection due to *B*.*pseudomallei* harboring BimA_Bm_ variant had significant associations with severe forms of the disease. Diabetes mellitus, septicemic melioidosis and infection due to LPSB genotype were independent risk factors for mortality. LPSB (74%) and LPSA (20.6%) were the prevalent genotypes causing infections. Both genotypes demonstrated temporal variations and had significant correlations with rainfall and humidity.

**Conclusion/Significance:**

Our study findings suggest that the pathogen specific virulence traits under the influence of ecological factors are the key drivers for geographical variations in the molecular epidemiology of melioidosis.

## Author summary

Amidst the ambiguity of its true incidence, melioidosis is gaining importance as an emerging infection in the Indian subcontinent. Variable virulence genes contributing to varied clinical presentation and the association of LPS genotypes provides key insights regarding the disease dynamics, host and pathogen specific determinants for disease presentations and outcomes among melioidosis patients in India. The study shows the divergence of Indian *B*.*pseudomallei* strains in context to LPS genotypes compared to isolates reported worldwide. The association of LPS B with mortality foreshadows the need for understanding the cascade of immune response triggered in the presence of different LPS genotypes.

## Introduction

Melioidosis is a fatal infectious disease caused by soil saprophytic bacterium, *Burkholderia pseudomallei*. Infection occurs mostly through the inhalation or percutaneous inoculation of the bacteria from contaminated soil or surface water. The disease manifests with diverse clinical presentations ranging from mild localized infection to fulminant sepsis. *B*. *pseudomallei* being a soil saprophyte, uses horizontal gene transfers as a mechanism for its persistence both in the environment and the host. It is possible that the virulence attributes of *B*.*pseudomallei* can significantly vary under the influence of regional environmental/ecological conditions, in turn leading to the occurrence of distinct clinical manifestations. Lipopolysaccharide (LPS) of *B*. *pseudomallei* is a well-known virulence factor that confers serum resistance and helps in evading host immune defenses during the early stages of infection. In this context, LPS is gaining recognition as a potential candidate for vaccine and diagnostic assay development [1,2]. Three genotypes of LPS namely A, B and B2 were reported previously with variations in their geographic distribution and ability to induce immune responses in animal models [3]. Burkholderia intracellular motility (BimA) and filamentous hemagglutinin gene (*fhaB3*) were reported previously as the significant variable virulence factors based on their geographic distribution and associations with clinical presentations [4].

Amidst the ambiguity of its true incidence, melioidosis is gaining importance as an emerging infection in the Indian subcontinent [5–7]. Sporadic cases reported from different parts of the country have shown assorted clinical presentations [7–10]. Distinct/novel sequence types of *B*. *pseudomallei* using multi-locus sequence typing(MLST) were previously reported from the south-western coastal part of India [11].This geographic region might presumably be one of the endemic hotspots of melioidosis in India reporting several cases [6,7,10]. Clinical isolates of *B*. *pseudomallei* from this region were genetically diverse from the Australian and Southeast Asian isolates and the prevalent sequence type (ST 1368) lacked significant association with any particular clinical presentation of the disease [11]. With this background, the present study documents the frequencies of variable virulence factors of *B*. *pseudomallei* and their association with clinical presentations of melioidosis and the temporal variations and influence of ecological factors on commonly occurring LPS genotypes of *B*. *pseudomallei*. Moreover, the host and pathogen-specific determinants for mortality are elucidated.

## Methods

### Study site and population

The present study was carried out at a tertiary care hospital with 2030 inpatient capacity that caters residents of southwestern coastal parts of Karnataka, India covering nearly 150–200 km radius of geographical area. This part of the country experiences tropical climatic condition with an annual rainfall of >4000 mm during June-October. Microbiological culture confirmed cases of melioidosis over a decade (2006–2016) were included in the study.

### Ethics statement

The study was approved by the Institutional Ethical Committee of Kasturba Hospital, Manipal. All the isolates were obtained from the archived collection and the identity of the patients was kept confidential.

### Study isolates

Isolates from blood culture in 97 bacteremic cases with (n = 50) or without (n = 47) other organ involvement and from other sites in 102 non-bacteremic cases were included in the study. For the extraction of bacterial DNA, QIAamp DNA mini kit (Qiagen, Hilden, Germany) was used as per the manufacturer’s instructions. Before inclusion, all the study isolates were confirmed as *B*. *pseudomallei* using a species-specific PCR targeting the TTSS1 gene cluster as described previously [12].

### Detection of LPS genotypes and variable virulence genes

Collectively, we aimed at detection of LPS genotypes (A, B and B2), BimA gene variants (BimA_Bp_ and BimA_Bm_) and *fhaB3*.

### Detection of LPS genotypes

Multiplex PCR assay simultaneously detected three different LPS genotypes LPS A, B, and B2 [3]. The PCR reaction was set at a final volume of 25 μl using JumpStartTaq Ready Mix (Sigma-Aldrich).Amplification was carried out in a Master cycler gradient (Eppendorf, Hamburg, Germany) with an initial denaturation at 95°C for 10 min, followed by 35 cycles of 95°C for 30sec, 59°C for 30 sec, 72°C for 30secand a final extension step of 72°C for 7 min. The oligonucleotide primers used in the present study and the expected amplicon sizes are tabulated in **Table 1**.

**Table 1:** List of oligonucleotide primers used to detect virulence genes of *B*. *pseudomallei*.

Virulence Determinants	Oligonucleotide sequence 5’-3’	Amplicon Size	Reference
BimA_Bm_	Bim_Bm_ F—AGCGCTTCGCGCATCTAC BIM_Bm_ R-CGCGTTAAACGCCGTACTTTC	104bp	4
BimA_Bp_	Bim_Bp_ F- GGAAGCTTTGGCGTGCATAT Bim_Bp_ R- CCCATGCCTTCCTCGACTAAT	60bp	4
*fhaB3*	*fhaB3* F-GACGCGGCACGTCTGATC *fhaB3* R-CGCGGATAAAACTCGGATTG	58bp	4
LPS A	wbiE_F-TCAAACCTATCCGCGTGTCGAAGT wbiE_R-TCGTCGTCAAGAAATCCCAGCCAT	195bp	3
LPS B	BUC3396F-AATCTTTTTCTGATTCCGTCC BUC3396RACCAGAAGACAAGGAGAAAGGCCA	93bp	3
LPS B2	BURP840_LPSb16-F- AACCGGGTAGTTCGCGATTAC BURP840_LPSb16-R-ATACGCCGGTGTAGAACAGTA	364bp	3

### Bim A detection

Both variants of Bim A, BimA_Bm_ and BimA_Bp_, were detected using previously reported PCR primers [4]. PCR reaction volumes and cycling conditions for the detection of BimA genes were similar to that of the LPS genotypes, except for a change in the annealing temperature to 56°C for 1 min. Presence of BimA_Bm_ and BimA_Bp_ were considered when amplicons sized 104 bp and 60 bp respectively were positive on 2.5% Agarose gel stained with 0.5% ethidium bromide.

### Filamentous hemagglutinin (fha) B3

*fhaB3* gene was detected using 0.3 uM of each forward and reverse primers to generate a 58 bp product [4]. Amplification was carried using similar cycling conditions as mentioned above for LPS genotypes detection. However, PCR for the detection of *fhaB3* was performed separately considering the similar size of the amplicons for both BimA_Bp_ and *fhaB3*.

### Clinical, epidemiological and meteorological data

Clinical and epidemiological data were documented in structured study forms. Monthly rainfall and relative humidity data for a period of six years (2010–2016) were obtained from the Indian Meteorological Department, Pune, India.

### Case definitions

Microbiological culture of blood and/ or other clinically relevant specimens was the mainstay for laboratory diagnosis of melioidosis. For analysis and reporting purposes the following case definitions were used in the present study:

- Bacteremic melioidosis: Patients with positive blood cultures for *B*. *pseudomallei*, with or without culture positivity of other clinically relevant specimens.
- Pulmonary melioidosis: Patients with clinical and radiological evidence of pneumonia / lower respiratory infection and isolation of *B*. *pseudomallei* from respiratory specimens and/or blood.
- Neurological melioidosis: Patients with clinical and radiological evidence of infection affecting brain tissue, meninges or spinal cord and isolation of *B*. *pseudomallei* from blood, cerebrospinal fluid or exudates.
- Septicemic melioidosis: Patients with features of sepsis (hyper/hypo-thermia, leukocytosis, hypotension, pulse rate >90/min, respiratory rate >18/min) and isolation of *B*. *pseudomallei* from any clinical specimen.
- Osteoarticular melioidosis: Patients with bone/joint infections such as osteomyelitis and septic arthritis, and isolation of *B*. *pseudomallei* from any clinical specimen.
- Localized melioidosis: Patients with skin and soft tissue (or any other single-site) infections that had no bacteremia and radiological or clinical evidence, suggestive of other organ involvement and isolation of *B*. *pseudomallei* from relevant clinical specimen.

### Statistical analysis

Descriptive statistical tools were used to determine the frequencies of categorical study variables. Pearson’s Chi-square test and Fisher’s exact test was used to check for the presence of any significant association of host and pathogen characteristics with individual clinical presentations. Risk factors for various clinical presentations and outcomes (in hospital mortality & Discharge Against Medical Advice) among the study population were determined using univariate analysis and step-wise multivariate logistic regression model (Backward LR) (SPSS, version 16). Time series analysis was used to decompose the trend, seasonal and residual components for the climate data and the LPS genotypes. Correlations of the climate data with the LPS genotypes was obtained using Pearson’s correlation coefficient. Generalized additive model was used to predict the LPS genotypes using the climate data. Analysis was carried out using R version 3.3.3. All values were considered significant with p≤0.05.

## Results

### Baseline demography and clinical data

**Fig 1:**
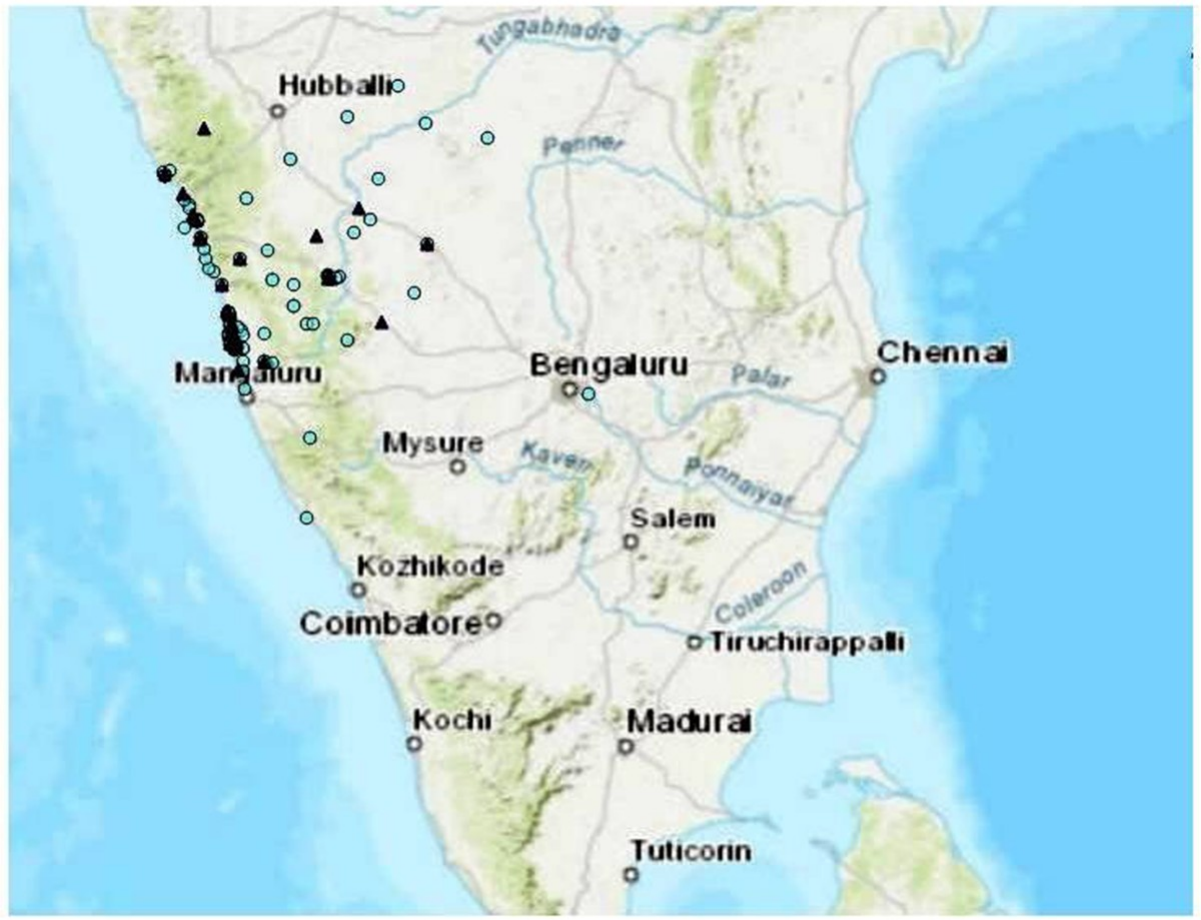
Geographical distribution of study patients and infecting LPS genotypes (Map generated using http://landsatlook.usgs.gov/.). LPSB genotypes: Light Blue circle, LPSA genotypes: Blue Triangles.

Mean age of our patients was 47.7±15.4 years, with an age range between 7–86 years. Of the 199 patients, 187 (94%) were from Karnataka and 12 patients (8 from Kerala and 4 from Goa) were from other states on the southwestern coastal part of India (Fig 1). Majority of the patients were males (153, 76.9%) and had the disease episode during monsoon (148, 74.3%).

A large number of patients (169, 85%) had either one or more of the severe forms of the disease, like bacteremic (97, 48.7%), pulmonary (71, 35.6%), septicemic (50, 25.1%) neurological (22, 11%) and others like osteoarticular and deep seated abscess (36, 18%). Amongst bacteremic patients, 47 (48.4%) had no other focus of infection as diagnosed clinically or radiologically. In septicemic melioidosis, 38 (76%) patients had bacteremia with or without evident foci and the rest 12 patients had osteoarticular or pulmonary forms of the disease. Localized form of the disease was observed amongst 30 (15%) patients.

### Association of host factors with clinical presentations

Diabetes mellitus (DM) (124, 62.3%) and renal dysfunction (27, 13.5%) were the common co-morbid illnesses. Three patients had malignancy, one had thalassemia, and none had HIV infection. Using univariate analysis, we observed that patients with renal dysfunction had 8.75 (Crude OR:8.75; 95% CI: 3.60–21.25; p<0.001) and 3.52 (Crude OR: 3.52; 95% CI: 1.41–8.77; p = 0.005) times more odds for developing septicemic and bacteremic forms of the disease respectively(Table 2).

**Table 2:** Association of demographical, seasonal and premorbid illnesses with clinical characteristics in study population.

Variables (N = 199)	Bacteremia (n = 97)	Pulmonary (n = 71)	Neurological (n = 22)	Sepsis (n = 50)	Localized (n = 30)	OA & DOA^#^ (n = 36)
**Age groups in yrs** <18 (14) 19–50 (99) >51 (86) p-value OR (95%CI)*	5 (35.7) 43 (43.4) 49 (57) 0.111 1.72(0.96–3.09)	5 (35.7) 35 (35.4) 31 (36) 0.995 0.97(0.53–1.77)	0 14 (14.1) 8 (9.3) 0.227 1.60(0.63–4.03)	2 (14.3) 24 (24.2) 24 (27.2) 0.530 1.21(0.62–2.33)	4 (28.6) 17 (17.2) 9 (10.5) 0.153 1.77(0.74–4.21)	4 (28.6) 18 (18.2) 14 (16.3) 0.541 1.14(0.53–2.46)
**Gender** Male (153) Female (46) p-value OR (95% CI)*	73 (47.7) 24 (52.2) 0.596 0.83 (0.43–1.61)	53 (34.6) 18 (39.1) 0.577 1.21(0.61–2.39)	17 (11.1) 5 (10.9) 0.963 0.97(0.33–2.80)	34 (22.2) 16 (34.8) 0.085 0.53(0.26–1.09)	22 (14.4) 8 (17.4) 0.617 1.25(0.51–3.04)	32 (20.9) 4 (8.7) 0.059 0.36(0.12–1.07)
**Season** Monsoon (148) Dry (51) p-value OR (95% CI)*	78 (52.7) 19 (37.3) 0.057 1.87(0.97–3.60)	56 (37.8) 15 (29.4) 0.279 0.68(0.34–1.36)	17 (11.5) 5 (9.8) 0.741 **0.83(0.29–2.39)**	41 (27.7) 9 (17.6) 0.153 1.78(0.80–3.99)	22 (14.9) 8 (15.7) 0.888 **1.06(0.44–2.56)**	23 (15.5) 13 (25.5) 0.111 1.85(0.86–4.01)
**Diabetes mellitus** Yes (124) No (75) p-value OR (95% CI)*	61 (49.2) 36 (48) 0.870 1.04(0.59–1.86)	49 (39.5) 22 (29.3) 0.146 0.63(0.34–1.17)	14 (11.3) 8 (10.7) 0.892 0.93(0.37–2.35)	36 (29) 14 (18.7) 0.102 1.78(0.88–3.58)	18 (14.5) 12 (16) 0.777 1.12(0.57–1.50)	26 (21) 10 (13.3) 0.175 0.58(0.26–1.28)
**Renal dysfunction** Yes (27) No (172) p-value OR (95% CI)*	20 (74.1) 77 (44.8) **0.005** **3.52(1.41–8.77)**	15 (55.6) 56 (32.6) **0.02** 0.38(0.17–0.88)	0 22 (100) **0.049** **0.84(0.79–0.90)**	18 (66.7) 32 (18.6) **<0.001** **8.75(3.60–21.25)**	3 (11.1) 27 (15.7) 0.773 1.49(0.41–5.29)	5 (18.5) 31 (18) 1.00 0.96(0.34–2.75)

^**#**^Osteoarticular and Deep Organ Abscess

* Crudes Odds Ratio is reported for all the variables

p value was calculated using Chi square or Fisher’s exact test.

### Frequencies of individual virulence factors and their association with clinical presentations

Majority of our study isolates belonged to LPS B (n = 147, 73.8%) followed by A (n = 41, 20.6%) and B2 (n = 11, 5.5%) genotypes. Amongst the variants of BimA gene, BimA_Bp_ and BimA_Bm_ were observed among 190 (95.4%) and 9 (4.5%) of the isolates respectively. Majority of the isolates were positive for *fhaB3*(190; 95.4%) and 181 (90.4%) isolates harbored both BimA_Bp_ and *fhaB3*genes. None of the three LPS genotypes had a significant association with clinical forms of the disease in our study population, as it was observed withBimA gene variants with neurological form of the disease(Crude OR: 12.72; 95% CI: 3.11–51.89; p>0.001)(Table 3).

**Table 3:** Association of lipopolysaccharide genotypes and variable virulence genes of *B*. *pseudomallei* isolates with clinical presentations in our study.

Variables (N = 199)	Bacteremia (n = 97)	Pulmonary (n = 71)	Neurological (n = 22)	Sepsis (n = 50)	Localized (n = 30)	OA & DOA^#^ (n = 36)
LPS A Yes (41) No (158) p-value OR (95% CI)	20 (48.8) 77 (47.7) 0.996 0.99(0.50–1.98)	17 (41.5) 54 (34.2) 0.386 1.36(0.67–2.75)	5 (12.2) 17 (10.8) 0.794 1.15(0.39–3.32)	11 (26.8) 39 (24.6) 0.492 0.76(0.35–1.64)	5 (12.2) 25 (15.8) 0.563 0.73(0.26–2.06)	9 (22) 27 (17.1) 0.471 1.36(0.58–3.18)
LPS B Yes (147) No (52) p-value OR (95% CI)	72 (49) 25 (48.1) 0.911 0.96(0.51–1.81)	52 (35.4) 19 (36.5) 0.880 0.95(0.49–1.83)	17 (11.6) 5 (9.6) 0.700 1.22(0.43–3.51)	36 (24.5) 14 (26.9) 0.728 1.13(0.55–2.33)	24 (16.3) 6 (11.5) 0.407 1.49(0.57–3.89)	26 (17.7) 10 (19.2) 0.804 0.90(0.40–2.02)
LPS B2 Yes (11) No (188) p-value OR (95% CI)	6 (54.5) 91 (48.4) 0.692 0.78(0.23–2.65)	2 (18.2) 69 (36.7) 0.334 0.38(0.08–1.82)	0 22 (11.7) 0.615 0.93(0.90–0.97)	3 (27.3) 47 (25) 1.00 0.88(0.22–3.48)	1 (9.1) 29 (15.4) 1.00 0.54(0.06–4.44)	1 (9.1) 35 (18.6) 0.693 0.43(0.05–3.52)
**BimA variants** BimA_Bp_(190) BimA_Bm_ (9) p-value OR (95% CI)	93 (48.9) 4 (44.4) 1.00 0.83(0.21–3.20)	69 (36.3) 2 (22.2) 0. 388 1.99(0.40–9.87)	17 (8.9) 5 (55.6) **0.001** **12.72(3.11–51.89)**	48 (25.3) 2 (22.2) 1.00 0.84(0.17–4.20)	27 (14.2) 3 (33.3) 0.138 0.33(0.07–1.40)	35 (18.4) 1 (11.1) 1.00 1.80(0.21–14.91)
***fhaB3*** Yes (190) No (9) p-value OR (95% CI)	94 (49.5) 3 (33.3) 0.344 0.51(0.12–2.10)	68 (35.8) 3 (33.3) 1.00 1.15(0.27–4.59)	21 (11.1) 1 (11.1) 1.00 0.99(0.11–8.34)	47 (24.7) 3 (33.3) 0.694 1.52(0.36–6.32)	30 (15.8) 0 0.360 0.94(0.91–0.98)	33 (17.4) 3 (33.3) 0.209 0.42(0.10–1.76)

^**#**^Osteoarticular and Deep Organ Abscess.Crudes Odds Ratio is reported for all the variables

p value was calculated using Chi square or Fisher exact test.

### Spatial and temporal variations of LPS genotypes

**Fig 2:**
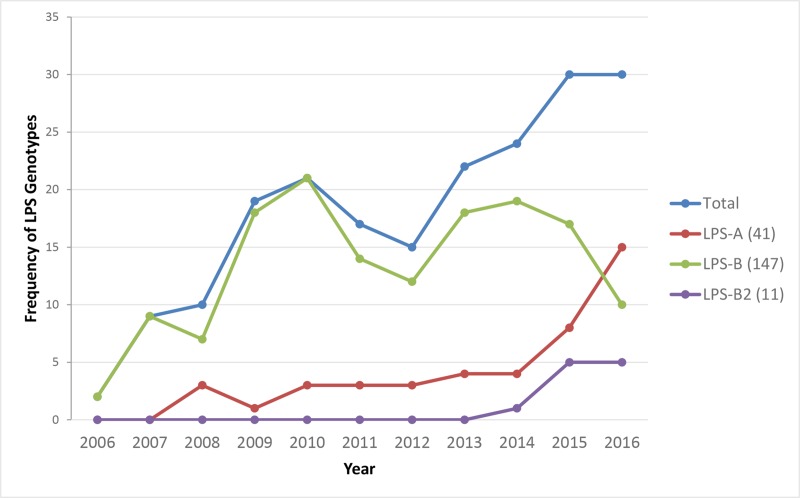
Year-wise distribution (2006–2016) of *B*.*pseudomallei* lipopolysaccharide genotypes causing infections in our settings.

Majority (73.8%) of the *B*. *pseudomallei* clinical isolates including all the 12 isolates from patients in the adjacent states belonged to the LPSB genotype (Fig 1). The prevalence of LPS A and LPS B genotypes were consistent throughout the study period (2006–2016), and LPS B2 genotype was observed only during the last three years (2014–2016). We noticed a steady decline of LPSB during the years 2014–2016 (79% in 2014, 56.6% in 2015 and 50% in 2016) in our settings. At the same time, there was a steady increase ofLPSA (16.6%, 26.6% and 33.3%) and B2 (4.1%, 16.6% and 16.6%) genotypes during the same duration (Fig 2).

### Influence of monthly rainfall and humidity on lipopolysaccharide diversity

**Fig 3:**
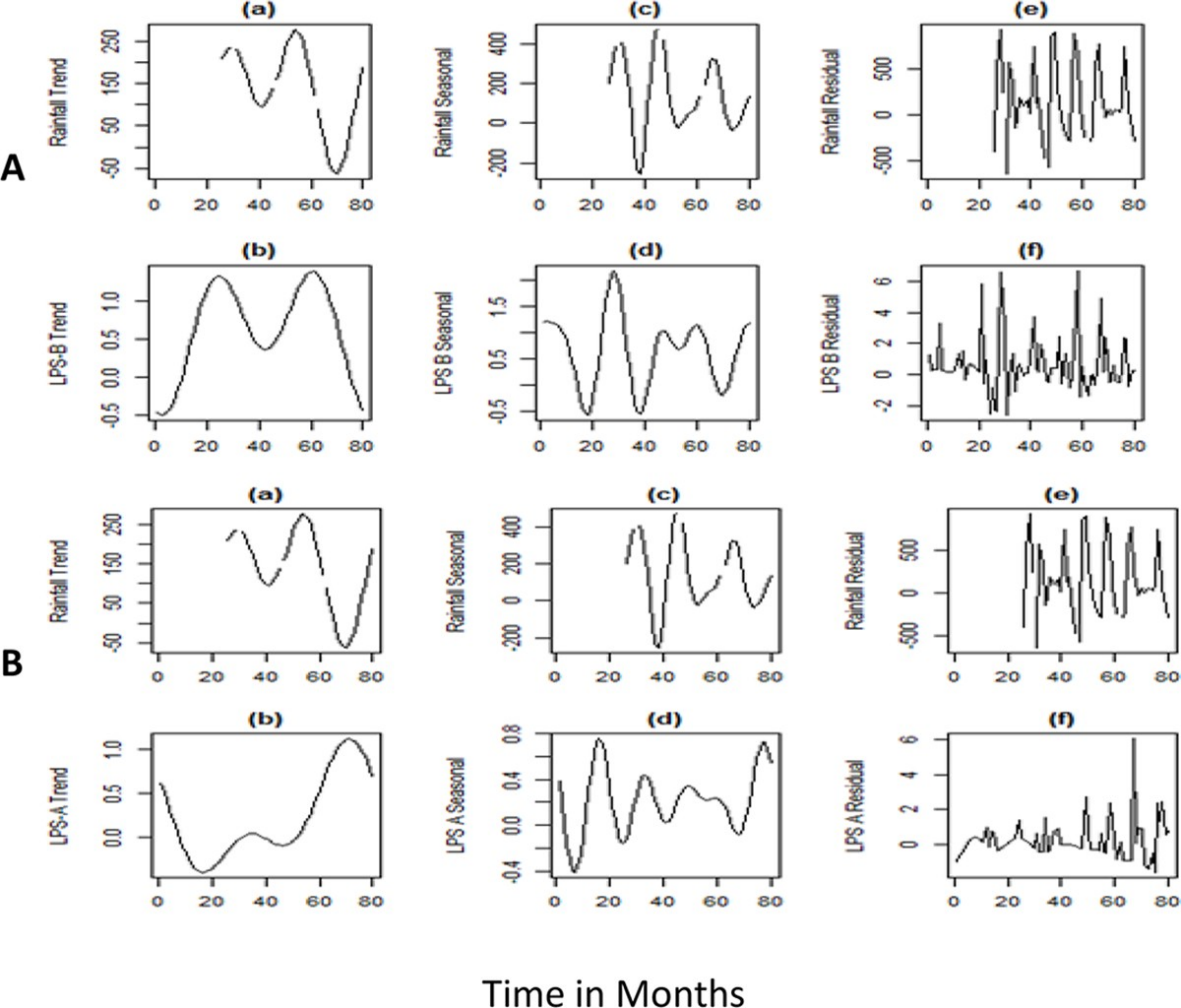
Fig A) shows reversal peaks for the trend and seasonal components of rainfall and LPS-A. B) shows an increase in LPS-B genotype with trend and seasonality. There is a lag period between the peak rainfall and the LPS-A and LPS-B genotypes.

**Fig 4:**
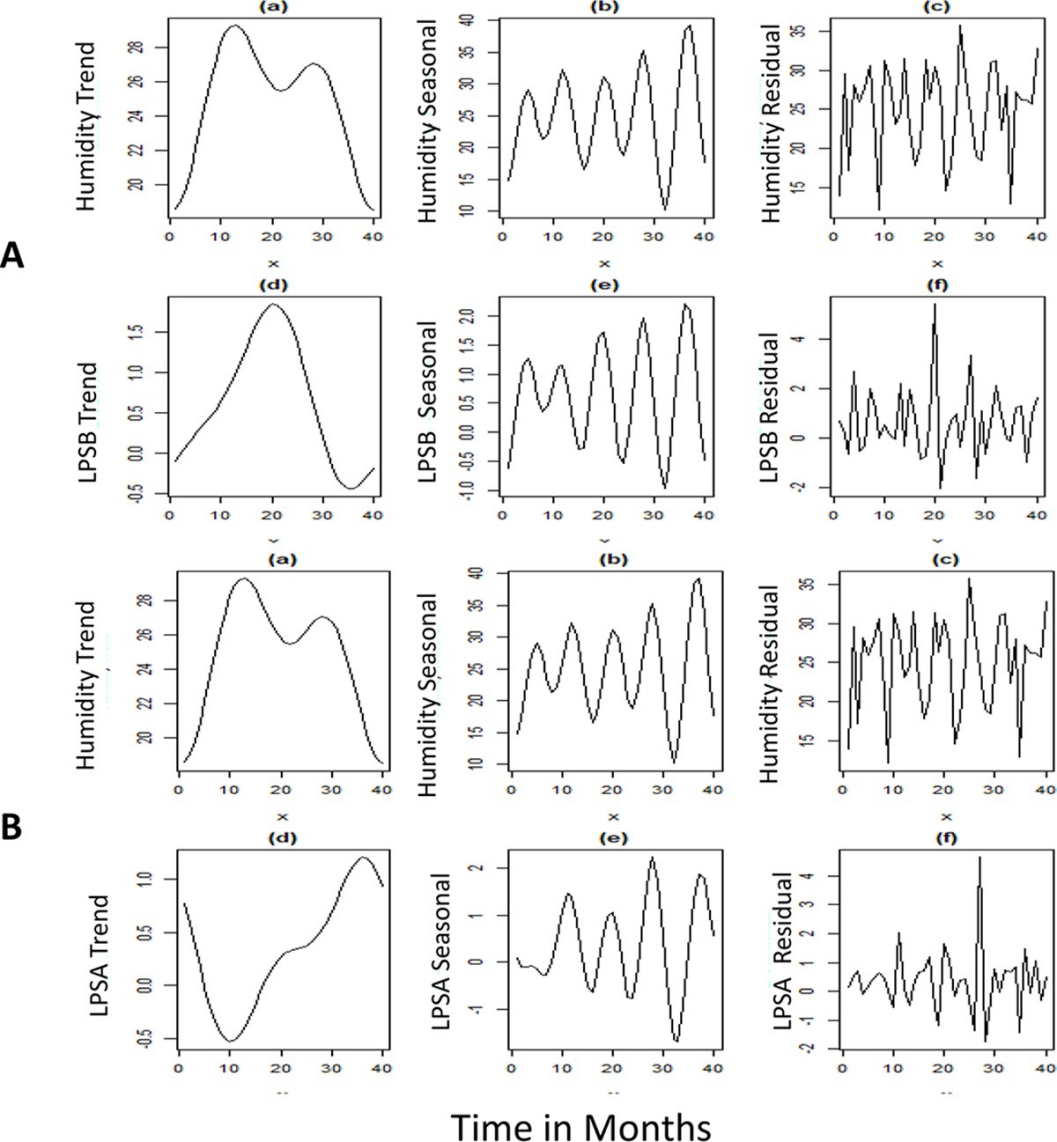
Fig A) shows reversal peaks for the trend component of humidity and LPS-A. B) shows an increase in LPS-B genotype with trend, seasonality and the residual component. There is a lag period between the peak humidity and LPS-B genotypes.

Monthly rainfall and humidity data for the years 2010–2016 was plotted against time and decomposed data for trend, seasonality and residual component were compared with LPSA and B genotypes. LPSA genotype showed a reversal peak for trend and seasonal components of rainfall, whereas rainfall had a significant increasing effect on LPSB genotype. There was a rise in LPSA genotypes observed over time, as compared to the LPSB genotypes. On omitting the seasonal trends, the residual component showed a positive effect on LPSB (p<0.001). The trend (p = 0.04) and seasonality(p = 0.01) for rainfall also showed a positive effect on LPSB genotype, whereas seasonality only had a positive correlation (p<0.001) for LPSA (**Figs 3 & 4**). Comparing the patterns of LPS genotypes with humidity, a positive correlation was observed between the seasonal component of humidity with LPSA (p<0.001) and LPSB (p = 0.003). A lag period was observed in the peaks of both LPSA and B genotypes compared to trend and seasonal component of rainfall.

### Treatment and outcomes

Among the 199 cases, 173 (87%) patients received melioidosis specific therapy. Of the 26 septicemic patients who did not receive pathogen specific therapy, 17 and 9 cases were with and without bacteremia respectively. Mortality due to melioidosis was observed among 51 (25.1%) patients. None of the patients with localized form of the disease had adverse clinical outcomes. Among the 50 patients with septicemic melioidosis, 23 (46%) succumbed to death. Case fatality rates were 14.5% (n = 25) among patients who received pathogen-specific therapy in comparison to 100% (n = 26) among those who did not receive the specific therapy.

### Host and pathogen specific determinants for clinical presentations and mortality

Renal dysfunction was an independent risk factor (after considering all the host and pathogen characteristics) for both bacteremic (Adjusted OR: 3.52 (1.41–8.87), p = 0.007) and septicemic (Adjusted OR: 9.70 (3.88–24.22), p<0.001) forms of the disease (**Tables 2 & 3**). Infection due to *B*. *pseudomallei* having BimA_Bm_ variant (Adj OR: 14.41 (3.16–65.58), p = 0.001) was an independent risk factor for neurological melioidosis in our study population. DM(26/51; 51%)[Adjusted OR: 2.37 (95%CI: 1.19–4.74), p = 0.014], septicemic melioidosis(32/51; 63%)[Adjusted OR: 2.74 (95%CI: 1.31–5.85), p = 0.007] and infection due to *B*. *pseudomallei* LPSB genotype (46/51; 90.1%) [Adjusted OR: 4.47 (95%CI: 1.16–12.20), p = 0.003] were independent risk factors for mortality in our study population.

## Discussion

*B*. *pseudomallei*, the etiological agent of melioidosis, demands no further negligence in view of its increasing geographical distribution, possession of numerous virulence traits and intrinsic resistance mechanisms to several antimicrobial agents. Severity of the infection and disease outcomes among patients significantly depend on the underlying host-factors, time for diagnosis/detection and befitting medical management. Few hostfactors such as DM, renal dysfunction and chronic alcoholism are well associated with poor disease outcomes among patients with melioidosis. *B*.*pseudomallei* possesses numerous proteins that play a pivotal role in the pathogenesis of the disease [4].Some genes that encode these proteins (conferring virulence) are known to be ubiquitously present among all the *B*. *pseudomallei* isolates [4].More recently, a study on the global evolution of *B*. *pseudomallei* reported geographically distinct genes/variants, conferring virulence among Australasian and Southeast Asian isolates [13]. However, there is a visible scant of whole genome sequencing data and the virulence attributes of *B*. *pseudomallei* isolates from regions outside northern Australia and northeast Thailand. Numerous studies have reported the influence of ecological factors on *B*. *pseudomallei* positivity in environmental niches [14, 15]. However, it is currently unknown if there is any influence of the ecological factors on the variable virulence genes of *B*. *pseudomallei* and whether these variations in the virulence gene profiles can lead to distinct clinical presentations and outcomes. Given this context, we report here the correlation of ecological factors in a geographical locality on infecting LPS genotypes of *B*. *pseudomallei* amongst patients.

Lipopolysaccharide of *B*. *pseudomallei* is an important virulence factor that facilitates the evasion of human immune responses during the early stages of infection. Monoclonal antibodies against the LPS of *B*. *pseudomallei* were found to reduce the severity of disease in animal models, thus implying the role of LPS as a potential vaccine candidate [1].Most intriguing finding from our study is that the majority (74%) of our patients were infected by the LPSB genotype of *B*. *pseudomallei*. This observation is in contrast with the findings from Thailand (2.3%) and Australia (13.8%), where LPSA was reported to be the prevalent infecting genotype [4]. Immunological responses and disease outcomes among animal models administered with LPS A and B types of *B*. *pseudomallei* were reported previously. While LPSA is known to confer serum resistance and grow in the presence of 10–30% of normal human serum [16], evidence from recent experimental and animal model studies suggest that LPSB is a more potent inducer of the pro-inflammatory cytokines and septic-shock [17]. In our previous study ST 1368 was the most common sequence type observed among 32 *B*.*pseudomallei* isolates [11]. Out of 14 ST 1368, 12 (85.7%) were LPSB,2 (14.2%) were LPSA and none were LPSB2. This finding suggests that the LPS genotypes can vary among isolates belonging to the same sequence type and thus making it difficult to ‘brand’ a particular ST as either a more or a less virulent one. In the present study, we did not observe a significant association of any of the three genotypes (A, B and B2) with any particular clinical presentation. We observed that infection due to LPSB genotype was an independent risk factor for mortality among our study population. However, we foresee the need for further validating this finding amongst patient populations from other geographic locations.

Expression of Burkholderia intracellular motility (BimA) protein is crucial in the pathogenesis of the disease. Among the two variants of Bim A known, BimA_Bp_ was the only variant reported among isolates from Thailand and other South Asian countries. On the contrary, isolates from Australia were reported to have both BimA_Bp_ and BimA_Bm_ variants [4]. Among our study isolates BimA_Bp_ variants were more commonly observed, but with no association with any particular clinical form of the disease. Presence of BimA_Bm_ was also observed in few (n = 9) of our isolates and had a significant association with neurological presentations. Similar association of BimA_Bm_ variant with neurological melioidosis was reported in Australian patients and more recently in a study using mice model [4, 18]. Filamentous hemagglutinin (FHA) is a surface protein of *B*. *pseudomallei* involved in adhesion to the host epithelial cells and formation of multinucleated giant cells [19].*fhaB3* is one of the three variable genes responsible for encoding the FHA protein, which was reported previously among 100% and 83% of the isolates from Thailand and Australia respectively [4]. Further, presence of *fhaB3* gene was reported in all the *B*. *pseudomallei* isolates obtained from Thai patients with bacteremic form of the disease and absence of *fhaB3* gene was reported to have a significant association with cutaneous melioidosis among Australian patients [4]. Presence of *fhaB3* gene was observed in almost 95% of our study isolates with no significant association with any form of the disease.

The epidemiology of melioidosis is characterized by environmental factors where rainfall plays a key role in transmission of the disease [20]. Majority of the cases in other endemic nations are known to occur during the monsoon when patients acquire the disease via inhalation or inoculation of the bacteria from soil and water. Occurrence of cases during the dry season, in many instances, is considered as a consequence of long latency and activation of the pathogen from latent foci [21]. In the present study, we observed that the LPSA genotypes had a reverse correlation with rainfall. This finding suggests the possibility of presence of LPSA genotype in dry environmental conditions, unlike the LPSB genotypes, which had a positive correlation with rainfall and humidity. However, we did not observe a significant increase in the occurrence of infections due to the LPSA genotypes during the dry season to support the assumption. Further analyzing the data, a lag period was observed between the occurrence of the cases and rainfall. The observed lag period could not be determined due to lack of enough data points and unavailability of weekly rainfall data, which remains as one of the limitations of our study. Temperature did not show any correlation with the infecting genotypes in our study, which can be attributed to the absence of striking variations in temperature through the year across the western coastal part of the country. Upon dismissing the seasonal trends, LPSB showed a positive correlation with the residual component, suggesting an influence of other environmental factors along with rainfall and humidity which needs further investigations.

Among the co-morbid illnesses observed in the present study population, renal dysfunction was found to be an independent risk factor for septicemic and bacteremic forms of the disease. DM was found to be an independent risk factor for mortality due to melioidosis in our settings, as reported in patients from Thailand and northern parts of Australia [22,23]. Strong association of DM with mortality was not surprising since 62% of the 169 patients with severe form of the disease were diabetic in our study cohort. However, DM did not have any significant association with any one particular form of the disease. Considering the high prevalence (16%) of DM amongst adult population residing in our settings [24], we foresee the need of more focused clinical studies to understand the disease outcome amongst patients with controlled and uncontrolled DM.

In our study cohort, though not statistically significant, we observed localized melioidosis amongst a higher proportion of patients with age <18 years (28.6%) in comparison with those with age >51 years (10.5%). Skin/soft tissue infections were the common clinical presentations among cases of localized melioidosis in our settings. These infections are more likely to occur in children due to their exposure to the bacteria while playing in water lodged fields during monsoon season. Higher frequency of localized, non-bacteremic cases were previously reported among Australian patients belonging to age group of <16 years [25]. Increased occurrence of localized form of the disease among patients of < 18 years of age can also be attributed to the lack of other predisposing factors responsible for the dissemination.

Put together, the present study reports few important host and pathogen-specific virulence determinants that have significant associations with clinical presentations and disease outcomes among Indian patients infected with *B*.*pseudomallei*. These findings can help in identifying high-risk cohort of patients for future studies aiming to understand the molecular pathogenesis mechanisms of the disease using integrated omics based approaches.
